# The value of bronchocele attenuation in pulmonary computed tomography in assessment of allergic bronchopulmonary aspergillosis in the background of cystic fibrosis: A cross-sectional study

**DOI:** 10.1016/j.amsu.2022.104892

**Published:** 2022-11-17

**Authors:** Mahsa Taherzadeh, Fatemeh Zamani, Neda Pak, Mohammadreza Modaresi

**Affiliations:** a Department of Radiology, Imam Khomeini Hospital, Tehran University of Medical Sciences, Tehran, Iran; b Children Medical Center of Excellence, Tehran University of Medical Sciences (TUMS), Tehran, Iran; c Advanced Diagnostic and Interventional Radiology Research Center, Tehran University of Medical Sciences, Tehran, Iran; d Pediatric Pulmonary Disease and Sleep Medicine Research Center, Pediatric Center of Excellence, Tehran University of Medical Science, Tehran, Iran; e Cystic Fibrosis Research Center, Iran CF Foundation (ICFF), Tehran, Iran

**Keywords:** Pediatric, Cystic fibrosis, Allergic bronchopulmonary aspergillosis (ABPA), Lung CT-Scan

## Abstract

**Background:**

There is no specific test in the definitive diagnostic approach to Allergic bronchopulmonary aspergillosis (ABPA) especially in the background of cystic fibrosis, but comprehensive and simultaneous clinical, radiological and serological examination will be the basis of ABPA diagnosis. The increasing in attenuation of bronchoceles in imaging has recently been proposed as a valuable diagnostic criterion.

**Purpose:**

The present study aimed to assess bronchocele attenuation in pulmonary CT scan of patients with complicated cystic fibrosis for diagnosis of ABPA.

**Methods:**

This cross-sectional study was performed on 74 consecutive patients aged 3–18 years suffering cystic fibrosis presented with exacerbation of pulmonary symptoms and were suspected of having ABPA. All were examined by 16 Slice CT Scan and the density of bronchoceles above 5 mm in diameter were measured in Hounsfield unit. The total serum IgE titer, skin prick test for aspergillus and anti-aspergillus IgG and IgE level were obtained for all subjects and both cutoff values of IgE level (>500 IU/mL and >1000 IU/mL) were considered as the criteria for ABPA diagnosis.

**Results:**

Considering IgE level of greater than 500 IU/mL and 1000 IU/mL as the diagnostic criteria, 24.3% and 10.8% had evidence of ABPA, respectively. Considering the two pointed diagnostic IgE ranges and based on the analysis of the area under the ROC curve, bronchocele attenuation could effectively predict the presence of ABPA with the best cutoff values of 37.25 (with a sensitivity of 70.6% and a specificity of 66.7%) and 40.00 (with a sensitivity of 85.7% and a specificity of 65.1%), respectively.

**Conclusion:**

The presence of bronchocele and an increase in its attenuation on CT scan will be diagnostic for the occurrence of ABPA.

## Highlights

• The bronchocele attenuation on lung CT scan can be diagnostic for ABPA in complicated CF.
• The increase in bronchocele attenuation on CT scan in association with increased serum IgE levels are important.
• Early diagnosis and rapid management of ABPA in CF are critical.

## 1 Introduction

Allergic bronchopulmonary aspergillosis (ABPA) is a potentially progressive disease that results from a hypersensitivity response to resistant Aspergillus fungal infection in the airways. Major advances have been made in understanding the role of allergic responses in the pathophysiology of such infections [1,2]. ABPA disease is more common in patients with asthma or cystic fibrosis, especially when associated with atopia [3,4]. Patients mostly present with symptoms that may be related to the underlying disease itself, so it is important to consider the occurrence of ABPA in such patients.

There is no specific disease test in the definitive diagnostic approach to ABPA disease, but comprehensive and simultaneous clinical, radiological and serological examination will be the basis of ABPA diagnosis [5,6]. When ABPA is suspected, total serum IgE level, Aspergillus IgG and IgE antibodies and a skin hypersensitivity test for Aspergillus infection should be performed. A positive skin test in the form of an IgE-mediated immune response is very sensitive for the diagnosis of Aspergillus infection but nonspecific for the diagnosis of ABPA [7]. In cases where the skin test is positive and total IgE levels are elevated, elevated levels of antibodies specific for Aspergillus fungal infection may help differentiate ABPA from Aspergillus allergy-induced asthma. Elevated serum eosinophil levels are helpful for the diagnosis but eosinophilia is completely nonspecific for the diagnosis of ABPA [8]. But in radiological evaluation, central bronchiectasis is the hallmark finding for ABPA, although in some cases ABPA has been reported without bronchiectasis [9]. In fact, the incidence of central bronchiectasis strongly suggests the occurrence of ABPA in cystic fibrosis. Overall, the diagnosis of ABPA in cystic fibrosis has been challenging and has always been debated due to its progressive nature. In general, the development of central bronchiectasis with pulmonary dysfunction, pulmonary tissue destruction with the production of concentrated mucus with severe and progressive clinical manifestations will often occur in the context of cystic fibrosis. In this regard, evaluation by serial CT scans along with changes in total serum IgE levels can also be very helpful. Also, a gradual decrease in pulmonary function in the pulmonary function test may strongly suggest cystic fibrosis with ABPA [[10], [11], [1][2]]. However, studies in different countries have shown that delayed detection of ABPA can take up to ten years after the onset of symptoms and, depending on the country, up to 50% of cases of ABPA may not be detected [13]. There is no gold standard for the diagnosis of ABPA, which makes it difficult to diagnose in patients with cystic fibrosis [14]. The radiological symptoms of ABPA are not specific, and these symptoms are seen in most patients with cystic fibrosis, whether they have ABPA or not [15,16].

No studies have been performed on mucus density and higher attenuation of bronchoceles in cystic fibrosis with ABPA. However, in recent case studies, patients with cystic fibrosis with higher concentrations of mucus and higher attenuation on CT scans have been reported [16]. The present study aimed to assess bronchocele attenuation in CT scan of lung of patients with complicated cystic fibrosis for diagnosis of ABPA.

## 2 Materials and methods

This cross-sectional study is fully complaint with the STROCSS 2021 criteria [17] and approved by the Chancellor of Research, Tehran University of Medical Sciences (IR.TUMS.VCR.REC.1397.027) [18]. The informed consent was approved by the Ethics Committee and obtained from patients before imaging. This study was conducted according to relevant guidelines and regulations in the Pediatric Center of Excellence, Tehran University of Medical Sciences, Tehran, Iran.

This study was performed on 74 consecutive patients aged 3–18 years suffering cystic fibrosis presented with exacerbation of pulmonary symptoms and were suspected of having ABPA in tertiary teaching hospital. The IgE titration was considered for all subjects. All patients were examined by 16 Slice GE HI Speed CT Scanner. The images were analyzed independently by two radiologists who were blind at the final diagnosis for Hounsfield bronchocele attenuation above 5 mm in CT scan and the density was recorded as bi-consensus. Patients were also evaluated and diagnosed for ABPA by specialists who were unaware of attenuation. Then, the patients whose ABPA diagnosis was definitive and those for whom this diagnosis was rejected were compared in terms of bronchocele attenuation. The following diagnostic criteria were considered for the diagnosis of ABPA in cystic fibrosis: minimum criteria including: 1) patients with cystic fibrosis,2)acute or sub-acute worsening of clinical signs for no other known reason, 3) total serum IgE greater than 1000 IU/mL, classic criteria and also above 500 IU/mL, as minimum criteria, 4) positive skin prick test with Aspergillus, 5)increased Aspergillus IgE and IgG antibodies,6)new or recent abnormalities on chest radiography or chest CT that not cleared with antibiotics and standard physiotherapy; and additional criteria including: 1)blood eosinophilia greater than 400 eosinophils/μL when patient is not on corticosteroids, 2)presence of precipitating Aspergillus antibody, 3)central bronchiectasis, 4) Aspergillus species-specific containing mucus plug [19]. For diagnosis, all major criteria must be present along with one minor criterion. In patients who showed more than one bronchocele on CT scan, the attenuation of all bronchoceles was recorded, but the highest attenuation was considered for comparison. Also, the bronchoceles that were blurred due to heart and lung movements and artifacts were excluded from the study. Patients' information was entered into a checklist that included the number of bronchoceles and the attenuation of each, the diagnosis of ABPA, as well as baseline variables including age, sex, IgE level, and the results of pulmonary function test were finally analyzed. Finally, the value of bronchocele attenuation for diagnosis of ABPA was assessed. In this study, both cutoff values of IgE (>500 IU/mL and >1000 IU/mL) were considered as the criteria for ABPA diagnosis.

Results were presented as mean ± standard deviation (SD) for quantitative variables and summarized in absolute and percentage frequencies for categorical variables. Normality of data was analyzed using the Kolmogorov-Smirnoff test. Categorical variables were compared using chi-square test or Fisher's exact test when more than 20% of bronchoceles with expected count of less than 5 were observed. Quantitative variables were also compared with *t*-test, or Mann *U* test. The ROC curve analysis was employed to assess the value of bronchocele attenuation for diagnosis of ABPA and in this regard it's the best cutoff point and related sensitivity and specificity were also determined. SPSS statistical software version 16.0 for Windows (SPSS Inc., Chicago, IL) was used for statistical analysis. P values of 0.05 or less were considered statistically significant.

## 3 Results

In the present study, a total of 74 patients with cystic fibrosis were included in the study. The mean age of patients was 7.66 ± 4.65 years, 35 cases (47.3%) were boys and 39 cases (52.7%) were girls. In terms of bronchocele detection, firstly, there was a high agreement between the two radiologists in terms of assessing the intensity of bronchocele attenuation (correlation coefficient equal to 0.833, p value less than 0.001).

**Fig. 1:**
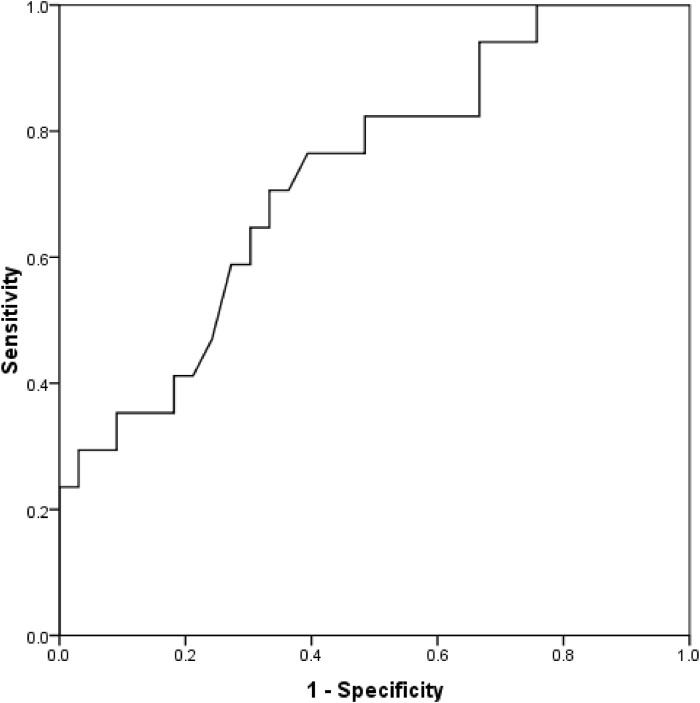
The area under the ROC curve in determining the diagnostic value of bronchocele attenuation in ABPA prediction (in terms of IgE >500 IU/mL).

Considering IgE >500 IU/mL as the diagnostic criteria, 18 cases (24.3%) had evidence of ABPA. Comparing baseline parameters between the two subgroups with and without ABPA (Table 1) showed higher mean age, higher outbreak of bronchocele as well as higher mean bronchocele attenuation, however the results in pulmonary function test were similar across the groups. The frequency of bronchocele in patients with and without ABPA was 94.4% and 58.9%, respectively. Considering the mean bronchocele attenuation evaluated by two radiologists, the mean attenuation in patients with and without ABPA was 54.23 ± 34.20 and 34.57 ± 13.09, respectively, which was significantly higher in the group with ABPA (P = 0.005). Based on the analysis of the area under the ROC curve, the assessment of bronchocele attenuation was a valuable parameter for predicting the presence of ABPA (area under the curve was 0.727, 95%confidence interval between 0.582 and 0.873, P value was 0.009). Accordingly, the best cut-off point for bronchocele attenuation in ABPA diagnosis was 37.25 with a sensitivity of 70.6% and a specificity of 66.7%. Based on the analysis of the area under the ROC curve (Fig. 1), IgE assessment was a very strong criterion for predicting the presence of ABPA (area under the curve was 0.995, 95% confidence interval between 0.984 and 1.000, P value was 0.001). Accordingly, the best cut-off point for bronchocele attenuation in ABPA diagnosis was 311.5 (with 100% sensitivity and 91.1% specificity). There was no significant correlation between bronchocele density and IgE level, FEV1 and FEV1/FVC (correlation coefficient equal to 0.175, P value equal to 0.630) in both groups with and without ABPA (Table 2).

**Table 1:** Underlying characteristics between two groups with and without ABPA.

Characteristics	Group with ABPA	Group without ABPA	P value
IgE >500 IU/mL			
Male gender	11 (61.1)	24 (42.9)	0.177
Mean age, year	10.94 ± 3.64	6.61 ± 4.47	<0.001
Mean serum IgE level	919.32 ± 323.94	124.67 ± 133.09	<0.001
Mean FEV1	54.32 ± 20.86	62.15 ± 24.55	0.367
Mean FEV1/FVC	76.88 ± 14.71	88.76 ± 22.96	0.076
Prevalence of bronchocele	17 (94.4)	33 (58.9)	<0.001
Mean bronchocele attenuation	54.23 ± 34.20	34.57 ± 13.09	0.005
IgE >1000 IU/mL			
Male gender	5 (62.5)	30 (45.5)	0.464
Mean age, year	11.62 ± 3.73	7.18 ± 4.54	0.010
Mean serum IgE level	1224.62 ± 71.77	208.60 ± 246.52	0.001
Mean FEV1	65.80 ± 25.02	59.12 ± 23.63	0.644
Mean FEV1/FVC	85.96 ± 11.61	84.94 ± 22.05	0.937
Mean bronchocele attenuation	69.21 ± 47.98	36.71 ± 14.10	0.001

**Table 2:** Correlation between bronchocele attenuation and underlying characteristics.

Characteristics	Correlation coefficient	P value
IgE >500 IU/mL		
Group with ABPA		
serum IgE level	0.249	0.335
FEV1	0.326	0.358
FEV1/FVC	0.175	0.630
Group without ABPA		
serum IgE level	0.064	0.723
FEV1	0.159	0.578
FEV1/FVC	0.246	0.397
IgE >1000 IU/mL		
Group with ABPA		
serum IgE level	0.300	0.063
FEV1	0.095	0.789
FEV1/FVC	0.122	0.656
Group without ABPA		
serum IgE level	0.201	0.196
FEV1	0.170	0.449
FEV1/FVC	0.291	0.189

**Fig. 2:**
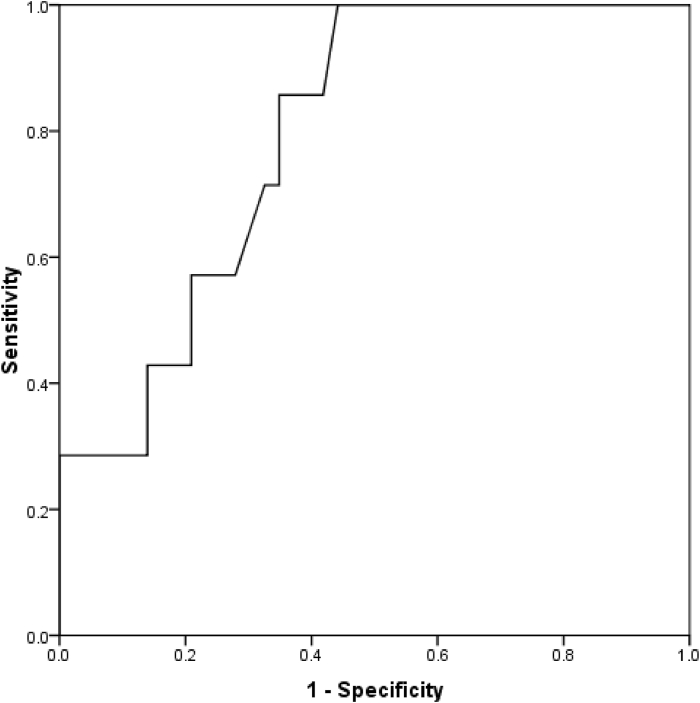
The area under the ROC curve in determining the diagnostic value of bronchocele attenuation in ABPA prediction (in terms of IgE >1000 IU/mL).

Based on the IgE level above 1000 IU/mL and Aspergillus IgG antibody, 8 cases (10.8%) had evidence of ABPA. Similarly, comparing baseline parameters between the two subgroups with and without ABPA (Table 1) showed higher mean age, higher outbreak of bronchocele as well as higher mean bronchocele attenuation without any difference in pulmonary functional parameters (Table 1). In terms of bronchocele detection, there was a high agreement between the two radiologists in terms of assessing the intensity of bronchocele attenuation (correlation coefficient equal to 0.935, P value less than 0.001). Considering the mean bronchocele attenuation evaluated by two radiologists, the mean attenuation in patients with and without ABPA was 69.21 ± 47.98 and 36.71 ± 14.10, respectively, which was significantly higher in the group with ABPA (P value was 0.001). Based on the analysis of the area under the ROC curve (Fig. 2), bronchocele attenuation assessment could effectively predict the presence of ABPA (area under the curve was 0.796, 95% confidence interval between 0.652 and 0.940, P value was 0.013). Accordingly, the best cut-off point for bronchocele attenuation in ABPA diagnosis was 40.00 (with a sensitivity of 85.7% and a specificity of 65.1%). We found no correlation between bronchocele attenuation and the baseline parameters of IgE level, FEV1 and FEV1/FVC in both subgroups with and without ABPA.

## 4 Discussion

In this study, we found first of all patients with cystic fibrosis, and based on the criteria, 24.3% (based on IgE above 500 IU/mL) and 10.8% (based on IgE above 1000 IU/mL) have the evidence of ABPA. This frequency was independent of the patient's gender (it was equally visible in boys and girls) but elevating with increase in age, which is completely consistent with previous studies. In previous studies, the occurrence of ABPA was mainly related to adolescents and was rarely observed at the age of less than 5 years. As another finding, the frequency of bronchocele in patients with and without ABPA was 94.4% and 58.9%, respectively, and the mean bronchocele attenuation in the group with ABPA was significantly higher. In fact, the presence of bronchocele and its higher attenuation can be considered an important parameter in the initial diagnosis of ABPA, which was confirmed by the analysis of the area under the ROC curve. In addition to the relevant findings, two important points were obtained in the present study. First, the increase in IgE levels, along with the other major criteria mentioned for the detection of ABPA is quite sensitive and specific. Therefore, the diagnostic role of bronchocele attenuation is complementary and cannot be considered as a means of ABPA validation alone. Second, in our study, pulmonary function testing (based on FEV1 and FEV1/FVC values) was similar in the two groups with and without ABPA. In fact, it seems that ABPA occurred in its early stages because only in the final stages of this complication, pulmonary fibrosis, obstructive pulmonary manifestations or pulmonary infiltration can be seen, which will be accompanied by a decrease in pulmonary function parameters.

There have been few studies on the incidence of ABPA in patients with cystic fibrosis and most studies have been done in the form of case reports. Similar to our study, in a study by Occelli et al., the presence of bronchi with a diameter of more than 5 mm had a high diagnostic value in differentiating patients with ABPA diagnosis and patients without ABPA [20]. However, in her study, no cutoff or sensitivity and specificity for this index was determined and evaluated. In a study by Amin et al., the view of multiple bronchoceles and central bronchiectasis indicated the diagnosis of ABPA. However, because the study included a description of an infected case, it was basically impossible to assess the diagnostic value of this view of multiple bronchoceles to diagnose ABPA [21]. Similarly, in a study by Laufer et al., in a majority of patients the appearance of multiple bronchoceles and central bronchiectasis was quite evident; again this finding was related to the description of an infected case [22]. According to a study by Osley et al., CT scan of the chest in patients with cystic fibrosis with ABPA showed higher attenuation in bronchocele [23]. Similar to our study, it was confirmed that an increase in bronchocele density could be diagnostic for ABPA, but the limitation of the study was that no cut-off point for bronchocele attenuation was proposed in the diagnosis of ABPA.

However, most studies (and quite similar to the present study) still emphasize the diagnostic nature of elevated serum IgE along with other clinical evidence such as skin testing for Aspergillus susceptibility. In the Zhang study, IgE was increased against Aspergillus antigen in 89% of patients and positive for Aspergillus antigen in 88% of patients. Also in the CT scan findings of the patients, 81% had bronchiectasis and 21% had mucoid impaction [24] and therefore the increase in IgE and skin test was still at the top of the diagnostic criteria for ABPA. Also, Marchant et al. showed that an increase in IgE levels above 500 IU/mL seriously indicates the diagnosis of ABPA in children with cystic fibrosis [25]. Therefore, the presence of elevated serum IgE levels along with Aspergillus skin test and confirmation of the relevant fungal infection can be diagnostic for the occurrence of ABPA in patients with cystic fibrosis, and the presence of pulmonary bronchocele with increasing attenuation of these marker will help to confirm the diagnosis.

## 5 Conclusion

As a final conclusion, a significant proportion of patients with cystic fibrosis will experience ABPA, which is a finding similar in both sexes but increases with age. The presence of bronchocele and an increase in its attenuation on CT scan will be diagnostic for the occurrence of ABPA, and along with other parameters such as increased serum IgE levels can be diagnostic for ABPA.

## Ethical approval

This research was carried out in compliance with the Helsinki Declaration and was approved by the Research Ethics Committee at Tehran University of Medical Science (IR.TUMS.CHMC.REC 1397.027).

## Please state any sources of funding for your research

The authors report no involvement in the research by the sponsor that could have influenced the outcome of this work.

## Author contribution

M.T. and F.Z Designed and performed experiments, analysed data and co-wrote the paper. N.P, M.R.M, F.Z Performed experiments and supervised the research. All authors contributed equally to the manuscript and read and approved the final version of the manuscript.

## Please state any conflicts of interest

The authors certify that there is no conflict of interest with any financial organization regarding the material discussed in the manuscript.

## Registration of research studies

1. Name of the registry:

Research Ethics Committee at Tehran University of Medical Sciences

2. Unique Identifying number or registration ID:

IR.TUMS.CHMC.REC 1397.027

3. Hyperlink to your specific registration (must be publicly accessible and will be checked):

https://ethics.research.ac.ir/ProposalCertificateEn.php?id = 23771&Print = true&NoPrintHeader = true&NoPrintFooter = true&NoPrintPageBorder = true&LetterPrint = true

## Guarantor

Fatemeh Zamani, M.D

Children Medical Center of Excellence, End of Keshavarz Blvd. Tehran, Iran

Tel: +98-9125013585 Fax: +98-2166581577

E-mail: doctorzamani@gmail.com

## Consent

The informed consent was approved by the Ethics Committee and obtained from patients before imaging. This study was conducted according to relevant guidelines and regulations in the Pediatric Center of Excellence, Tehran University of Medical Sciences, Tehran, Iran.

## Provenance and peer review

Provenance and peer review not commissioned, externally peer-reviewed.
